# Participatory Research Revealing the Work and Occupational Health Hazards of Cooperative Recyclers in Brazil

**DOI:** 10.3390/ijerph10104607

**Published:** 2013-09-27

**Authors:** Jutta Gutberlet, Angela M. Baeder, Nídia N. Pontuschka, Sonia M. N. Felipone, Tereza L. F. dos Santos

**Affiliations:** 1 Department of Geography, University of Victoria, P.O. Box 3060, STN CSC, Victoria, BC V8W 3R4, Canada; 2 CAPES-Visiting Professor, University of São Paulo, USP, Avenida Universitária, 308, São Paulo, SP, CEP: 05508-000, Brazil; 3 Department of Biology, University Foundation Santo André, FSA, Avenida Príncipe de Gales, 821, Santo André, SP, CEP: 09060-650, Brazil; E-Mail: baedpint@yahoo.com.br; 4 Faculty of Education and Department of Geography, University of São Paulo, USP, Av. Da Universidade, 308, CEP: 05508, São Paulo, SP, Brazil; E-Mail: nidia@usp.br; 5 Reference Centre on Occupational Health, Rua João dos Santos Werneck, 51, São Paulo, SP, CEP: 04437-110, Brazil; E-Mail: sonia.felipone@gmail.com; 6 FUNDACENTRO, Ministry of Labour and Employment of Brazil, Rua Capote Valente, 710, São Paulo, SP, CEP: 05409-002, Brazil; E-Mail: ferreira@fundacentro.gov.br

**Keywords:** informal recycling, recycling cooperative, occupational health, environmental education, action research, participation, Brazil

## Abstract

Although informal waste collectors are sometimes organized in cooperatives, their working conditions remain extremely precarious and unsafe. The paper discusses the findings of action oriented, participatory qualitative research with several recycling groups in the metropolitan region of São Paulo, Brazil. During workshops with the recyclers mapping, acting, and drawing methods helped reveal health hazards from collection, separation and transportation of recyclable materials. Major health problems relate to chemical and biological hazards, musculoskeletal damage, mechanical trauma and poor emotional wellbeing. The recent federal legislation on solid waste management opens new avenues for the inclusion of recycling cooperatives in selective waste collection. Nevertheless, we express the need to consider the distinctive characteristics and vulnerabilities of recycling groups, when developing safer work environments in these social businesses. We also suggest that the workspace be ergonomically organized and that public awareness campaigns about selective waste collection are conducted regularly to increase the quality of source separation. The introduction of electric hand pushed carts can further reduce health strains. This research has produced a better understanding of the work of the recyclers and related health risks. The interactive qualitative research methodology has allowed for the co-creation and mobilization of specific knowledge on health and safety in recycling cooperatives.

## 1. Introduction

Selective waste collection, material separation and recycling are widespread activities conducted informally or organized through associations and cooperatives. In most countries of the global south approximately 1% of the population is involved in this form of resource recovery [1,2]. Here most of the informal recycling still happens on an individual basis, with the collection of recyclable material from the household waste disposed in the street, from offices or businesses or, as worst case scenario, salvaged from landfills and irregular dumps [3]. These informal recyclers or collectors, in Brazil called *catadores*, often separate and store the materials at home, adding yet another facet of hazardous health implications to their lives.

In many countries and particularly in big cities in the global north, informal selective waste collection is also a reality [4,5]. Here the activity involves primarily the recovery of beverage containers, but also scrap metals, plastics, paper and cardboard or specific items such as car batteries, wires or electronic and electrical waste. Most of these workers are socially and economically excluded and live at the margin of society, also subject to poverty related health impacts.

Organized selective waste collection is more prominent in the global south [2,3]. In Brazil, for example, *catadores* organize into cooperatives, sometimes with the support of government or non-governmental organizations, working in the collection, separation and commercialization of recyclable materials. The level of organization, the availability of space and infrastructure assisting the work process (such as presses, forklifts, tables, computers, *etc*.), as well as the administrative and business skills vary greatly among the groups, as does the number of cooperative members. Most recycling cooperatives have a high turnover of their workers, which challenges the continuity and programming and for which reason human development and capacity building activities need to be continuous.

The current study was developed with several recycling cooperatives supported through an international research and community outreach cooperation (the *Participatory Sustainable Waste Management* project (PSWM) [6], by its participants also called the *Brazil-Canada project*), of which the authors were part since 2006. This project was aimed at strengthening recycling groups in greater metropolitan São Paulo, increasing the dialogue between *catadores* and government, supporting the design of adequate and inclusive solid waste management public policies and among several other research objectives the investigation of occupational health and hazards in recycling cooperatives. We were able to conduct the current study because of the long established trustful relationship between members of the project and of recycling cooperatives. The inclusive approach of this larger research collaboration, based on participatory and deliberative project management methods, enabled us to work closely with some of the members of recycling groups in this present research on occupational health with six recycling cooperatives in Greater São Paulo.

In the present study, the aim was to collectively generate knowledge on the working conditions and possible hazards and risks linked to the collection, classification, manipulation and transportation of the materials separated for recycling purposes. The research process was participatory and action oriented and therefore in itself rich in generating knowledge and stirring social change among the participants. *Catadores* still suffer from widespread stigmatization and social/economic exclusion. Being able to tell their stories, listen to the others’ experiences and question the causes for economic exclusion and social stigmatization are important parts in empowerment of individuals. Our research took a qualitative approach, confirming and valuing previously neglected knowledge and ultimately contributing to a better understanding of the complex social, cultural, economic and political conditions that shape the work of these people. Our methodology was based on participatory, action oriented and community-based research [7,8,9,10,11,12] grounded in an epistemology of knowledge co-creation [13,14,15] and feminist theory [16]. The research process of inquiry was combined with capacity-building strategies, helping overcome knowledge gaps, and empowering and making participants visible as agents for social change. We were guided by the ideal of understanding the research process as a contribution to the development of skills, knowledge and capacities that facilitate the use of the results by the participants themselves [17].

We will begin by discussing the research process in detail and highlighting major results. The research has evidenced and co-created important knowledge on the occupational health situation as well as on environmental education directives involving organized informal recyclers. The multifarious health and safety issues related to the different work activities in the recycling cooperatives will be discussed in detail as well as some of the suggested solutions for some of the problems. Specific attention will be given to the contributions and possible impacts of recent workers’ health legislation.

## 2. Participatory Methodology Revealing Living and Working Conditions of *Catadores*

The major objective of the *Participatory Sustainable Waste Management* (PSWM) project was to contribute to the consolidation and strengthening of recycling cooperatives as active players in selective waste collection and recycling activities [6]. The current research involved a multi-disciplinary and multi-institutional work team, composed of the five authors (three university professors, one researcher on occupational health from FUNDACENTRO and one occupational health therapist), one support person and two graduate students. This research followed the request expressed by the recyclers of the PSWM project to initiate actions with a focus on environmental education and health.

Resource recovery performed by *catadores* is multifaceted and requires an interdisciplinary lens. Selective waste collection improves the quality of the urban environment by removing waste and creating educational opportunities by explaining and interacting with the general public and administrators on themes related to the multiple dimensions of public cleaning, environmental management and on how selective waste collection promotes social inclusion. Nevertheless, the collection and sorting of solid waste exposes the workers to many different health risks. For those *catadores* who work informally outside of a cooperative, risks are even larger [18,19,20,21]. The literature on occupational health problems related to the collection and separation of recyclable materials distinguishes chemical and biological risks by contaminated materials, problems related to posture, emotional well-being and injuries by accidents [22,23,24,25].

Over the last decade, public waste management concepts integrating selective waste collection with social inclusion have become more widespread in Brazil. These approaches, however, have brought up challenges as a result of the lack of public environmental awareness and education as well as the absence of a continuous dialogue between the recyclers, the government, and the general public. The need to improve the health conditions of these workers is evident, given the fact that this professional activity is still performed without specific occupational health regulations, as has been achieved by other professional categories. Therefore it is essential to study the work environment of the recyclers and the health problems related to this activity, identified by the protagonists themselves.

Our initial challenge in this research was to define an appropriate methodology that would provoke a critical reflection on occupational health risks from selective waste collection, rejecting the understanding that these risks are a natural given. Our methodology had to contribute to the awareness building process of the recyclers, in the sense of Paulo Freire’s concept of “*conscientização*” [26] and had to promote a collective construction of better environmental and occupational health conditions.

In the waste management context, we understand environmental education as a form of education, which promotes conceptual change in the behavior and way of life with regards to waste problems (from generation to final disposal of waste). It was thus essential to identify the material components of the recycling chain, the social actors involved in consumption and discarding, and the role of organized recyclers—specifically their cooperatives. Environmental education, as in education in general, is a political act that nurtures citizenship and autonomy, allowing for the recognition of conflicts of interest, and in this context, also the perception of possibilities of actions. Within the perspective of cooperative selective waste collection, environmental education happens when learning through the recyclers’ experiences of environmental management, as conceptualized by Loureiro [27]. In waste management, educational actions extend the power of action, the exercise of citizenship, and the promotion of both social transformations and change in human-nature relationships [28]. We assumed that reflections on the issues raised during the meetings with the recyclers would contribute to their empowerment and create the potential to further engage the dialogue with the population. We are aware that the quality of this dialogue allows for important change that decreases the health risks of the recyclers, because awareness will result in better household separation of recyclable material and thus reduce some of the health risks of the recyclers.

Active participation was the most fundamental methodological principle applied in this study and it was essential for the construction of a collective understanding of the praxis experienced by the recyclers in their daily life. Our research process was participatory and began with collectively defining the selection criteria for the representatives from the cooperatives to be involved in the workshops. The dynamic of the workshops and of the meetings in the cooperatives helped to socialize and begin discussions of the research results in the cooperative.

**Plate 1:**
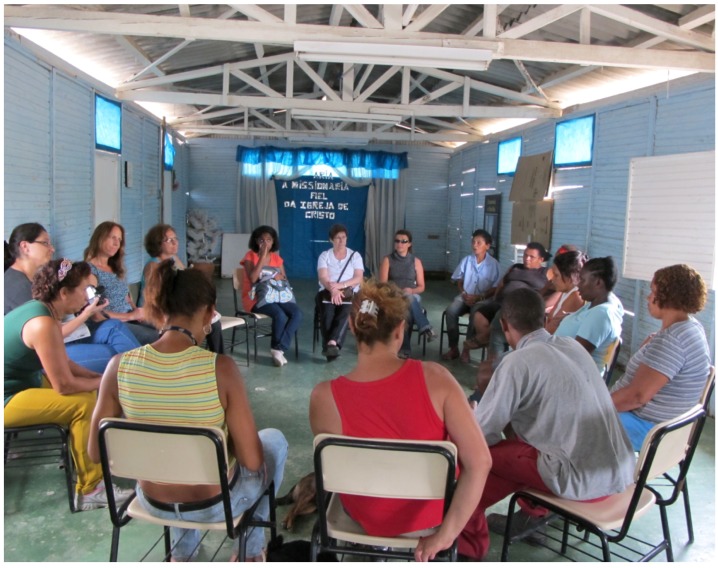
Introductory meetings with recyclers from COOPERCOSE in São Paulo (15 February 2011).

The research was organized into three phases: mobilization, workshops, and feedback sessions. During mobilization our group presented the idea and objectives of the action oriented study at the six recycling cooperatives and invited their members to choose two representatives to participate in the workshops and to act as knowledge transmitters between the research group and the cooperative (Plate 1). At the beginning of the workshops, *ice-breaking* activities were conducted to support an open and trustful learning environment. The workshops (conducted at the premises of FUNDACENTRO, a national research centre on occupational health and safety) involved brainstorming and active learning, applying collective mapping, acting, and drawing methods focusing on possible risks and health hazards as well as respective strategies to overcome these during the work phases of collecting, separating, and manipulating recyclable materials.

During the workshops, the cooperative members mapped key health aspects related to the working conditions, based on their practical knowledge. This information formed the basis of our discussions and guided the definition of priorities in our action-oriented research. During the mapping activity, the predominant linkages between health conditions and the current historical context of selective waste collection, as well as the diversity of human and physical conditions were discussed. Particular political local contexts that differentiated each one of the municipalities were also raised.

The collective involvement of the recyclers ensured the surfacing of valuable information on the quality of life, the state of their health, and on some characteristics of the municipal solid waste management system. Interactive dynamic activities facilitated by the group of supporters were conducive to voicing some of the problems the recyclers had experienced or were currently facing, as well as the specifics of their work in relation to the exposure to risks and dangers.

Neither research participants nor the cooperatives themselves were understood as “study objects”, but were instead valued as active participants in the research process on their living and working conditions. The research allowed for the recognition of the links between the historical, political, economic and environmental context of the country. The everyday practice and immersed knowledge of the political dimension of these groups of cooperative members and supporters/researchers consolidated a commitment towards promoting empowerment, autonomy and the ability to overcome the oppression of the hegemonic power so present in capitalist modes of production. Respect for the knowledge of the participants, the co-construction of new knowledge and its systematization for collective ownership were fundamental principles in our methodology. The constant reflexive concern of the research group involving *catadores* and applying participatory research techniques has been substantiated and corroborated by authors such as Rodrigues Brandão, Fals Borda, Thiollent and Barbier [29,30,31,32,33], among others.

Brandão believes that at all times the collective reflection on reality is present and that people take ownership of knowledge and write their own social history. *Learn to rewrite history through your own history. The social actor that researches is a kind of a person that serves. These kinds of people are allies and are armed with scientific knowledge that has always been denied to the public and to those for whom the participatory research should be yet another instrument of popular conquest. Participatory research means researcher and researched alike are actors of the same work, although with different contexts and tasks* ([9]: p. 11) (translation by the authors).

In the design of participatory research, the data produced by the bases must return to them together with new information that serves them directly as a reference for interventions and actions. In our study this return happened during the workshops and meetings, where a direct link was established to collective decision-making regarding necessary actions and through the process of systematizing the collected information. From the point of view of actions and interventions in the daily life and at the workspace of the cooperative members, our objective was to transform everyday practices through the constant dialogue during the meetings and workshops. In this respect, our research is action oriented and qualitative in nature, resonating with the idea that: *(t)here is a growing gap between the knowledge used in solving real problems and the knowledge used only in a rhetorical or symbolic sense in the cultural sphere* ([33]: p. 9) (translation by the authors). Thiollent reinforces the fact that the practical problems of everyday life, while being detected, already point to the beginning of the path to be travelled towards a solution. In this author’s writings, he shows that qualitative research also bears dangers, but that these can be controlled or minimized by a well-defined methodological stance, appropriate to the situation under consideration.

When working with cooperative members and informal recyclers who have lived in a situation of exclusion, interactive research dynamics allow for deepening the change of subjectivities, of different languages, strengthening the self-esteem and the potential of the recyclers [28]. The exchange of experiences with these methodological principles allowed for the strengthening of the participants’ identity, which is essential for meaningful participation.

The research process was further supplemented through theory of Pedagogic Psychodrama (*Psicodrama Pedagógico*) [34], which systematizes three steps in relation to knowledge construction. It begins with the emotional or intuitive approach of the participants in relation to the theme, followed by a rational or conceptual approach, and concluded with a functional approximation towards the discussed concepts. Thus, the work is based on the perceived or felt knowledge of the group about the theme under discussion, followed by a reflective process, until reaching the desired concept, which corresponds to the new, collectively built knowledge. The approach values cooperative learning. It expresses feelings and promotes participation, interaction, and the sharing of experiences.

**Plate 2:**
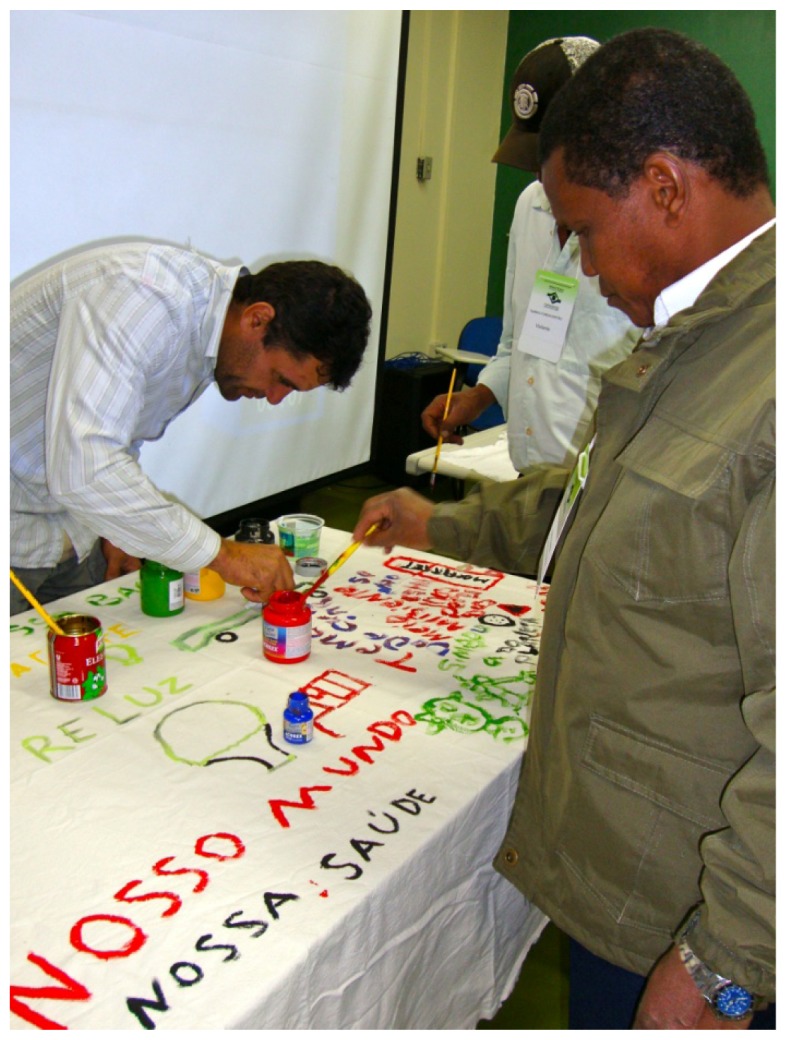
Workshop with drawing activity (9 June 2011).

**Plate 3:**
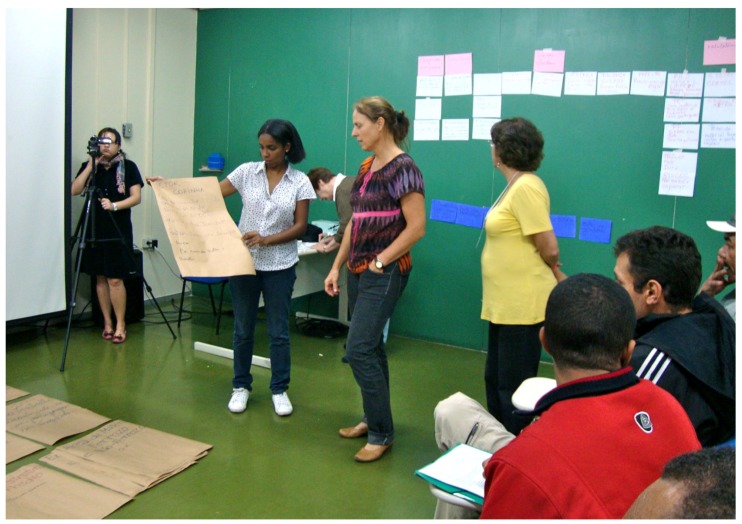
Collective knowledge generation during the workshop (26 May 2011).

The richness of the interactions during the workshop and meetings became transparent with a plurality of expressions, through oral and body language, plastic, mythical and musical expressions, depicting imaginations besides numerous elements that reference different readings of the world, of the systematized knowledge and of popular wisdom (Plate 2 and Plate 3).

The comprehension of the educational processes requires the understanding of these interactions, inside of which, according to Burnham: *… inter-subjectively the subjects build themselves, the knowledge already produced, and the knowledge newly created, their relations with each other and with their reality, as well as, by the action (both in the subject’s individual and social dimension), transform this reality by way of a multiple, cyclic process that contains, in itself, both the face of continuity and the construction of the new* ([32]: p. 37) (translation by the authors).

With finalizing the workshops on environmental education and health, we began another step in the research process that we call *Feedback*, which meant presenting the research results to all members of the six cooperatives and engaging in a collective reflection on new processes of improvement in workspaces and the interactions between the workers. A meeting was held in each one of the six cooperatives that participated in the study to present and to discuss the results achieved through this process of knowledge co-creation. The key aspects discussed were related to the urgency of strengthening the group’s perception of occupational health risks, the value of the exchange of experiences between different recycling cooperatives, the need to involve other social agents to address solutions for the occupational health problems, the importance of ensuring dialogue among recycling groups, and the need to promote collective forms for the assessment of health issues in the workspace. Later, an educational pamphlet summarizing the research process and the results was distributed to the participants as well as to other recycling cooperatives in the region.

## 3. Research Findings about Core Health Concerns of *Cooperative Recyclers*

Four fundamental questions linked to occupational health and environmental education of informal and organized recycling guided our reflections and supported the thematic structure of the workshops and meetings at the cooperatives. What are the major health related problems in the work of *catadores*? What does it mean to be a *catador*? How do the *catadores* work? What is the productive chain for recyclable materials?

The following table (Table 1) summarizes the key findings in this inquiry, which will then be discussed in detail. Most of these problems have also been identified as risks by other studies as highlighted in the bibliographic revision on occupational health and risks of informal and organized recyclers conducted by Binion and Gutberlet [22].

**Table 1:** Key health hazards and risks identified during the workshops.

Health hazards	Description
Chemical	Residues in packaging, such as: toxic cleaning products containers, cement bags, *etc*.
Biological	Contact with fungus and bacteria accumulating in contaminated packaging, food scraps mixed with recyclable materials, infections due to disease vectors such as pigeons, rats, insects, *etc*.
Physical	Insufficient lighting, lack of ventilation, irregular floor surfaces or damaged floor pavements, lack of roof cover or damaged roof and water leaks.
Accidents	Accidents during the collection in the street (car accidents); accidents in the cooperative: e.g., loss of limbs when operating the press, unstable piles, unsafe surfaces, cuts due to sharp instruments, glass, metal, sharp paper and plastics mixed with the materials.
Ergonometric	Inadequate posture due to lack of correct infrastructure in the collection, separation and processing of recyclable materials, lack of fresh air circulation, insufficient lightning, unsafe work organization.
Emotional vulnerabilities	Social stigma, stress, depression, anxiety, power imbalances, dependencies (drugs, alcohol).

*Catadores* themselves consider their work as one of heavy lifting, while others perceive it as dirty, dangerous, or an activity that goes back to the idea of rag picking during the Middle Ages. Much more than rag picking, *catadores* are responsible for collecting much of the reusable and recyclable material and represent an important link in the production chain of recyclables. Recyclers are resource recoverers. However, informal recycling as it is currently performed often means an activity, which is painful, dangerous, and unhealthy to those involved.

Every day approximately 150,000 to 160,000 metric tons of municipal solid waste is collected in Brazil ([35]: p. 44). On average the solid waste in Brazil contains 57.4% organic matter, 16.4% plastics, 13.2% cardboard, 2.3% glass, 1.6% ferrous material, 0.5% aluminium, 0.5% inert material and 8.1% other materials. Official census data from 2000 identified 445 municipalities as having selective waste collection and there were approximately 21,500 informal recyclers working at waste dumps [36]. In 2008, 994 municipalities already had a selective waste collection program in place; 65% of which practiced the collection in partnership with organized recyclers and in the remaining municipalities, recycling cooperatives conducted the separation and collection independently [36]. Cunha and Borges report “*in Brazil there are almost a million people who work in resource recovery and recycling, and consequently, are in favour of the environment, which is about 15% of the economically active population*” ([37]: p. 16) (translation by the authors). Estimates about the total number of *catadores* in Brazil vary from 500,000 [38] to 1 million [39]. These workers carry out part of their work in the open and do not have the facilities to satisfy their basic physiological needs, nor do they have access to drinking water and food, and they often collect recyclables during long hours.

The workshops revealed the exposure of the recyclers to all sorts of different risks during the various phases of their work: collection, reception, transportation of the materials, classification (specific material separation for cardboard, ferrous materials, soft and hard plastics, different sorts of glass, leather and textiles, furniture), pressing and weighting, storing, transporting and marketing. They are also exposed to risks due to the unsatisfactory sanitary conditions in their work environment, including the toilet area, change rooms, and refectory, which in some of the cooperatives were characterized by a lack of hygiene and comfort.

Throughout the collection phase, the bodies of these recyclers perform numerous movements of lifting up and down to the cart or truck. When operating hand carts they have to push heavy weights of the material collected, risking straining of the muscles and spine, or causing pain in the arms, legs, and the column.

During the door-to-door collection, the recyclers are exposed to additional risks, with the inadequate disposal of recyclable materials by some households. For example, sharp objects within the material can cause cuts on hands and arms. Still related to the collection phase, the recyclers mentioned irregular meal times as a health issue, particularly when working independently in the street. With regards to the quality of the material collected, in addition to cutting and perforating objects, it was repeatedly mentioned that the material comes dirty, sometimes with food waste, and thus exposing them to biological and/or chemical contamination. It is worth noting that access to personal protective equipment such as gloves and mouth protection is rare in this activity, and even if available, the recyclers do not always wear the equipment. Other studies and our own observations during many years of research on informal and organized recycling are consistent with these results [40,41].

**Plate 4:**
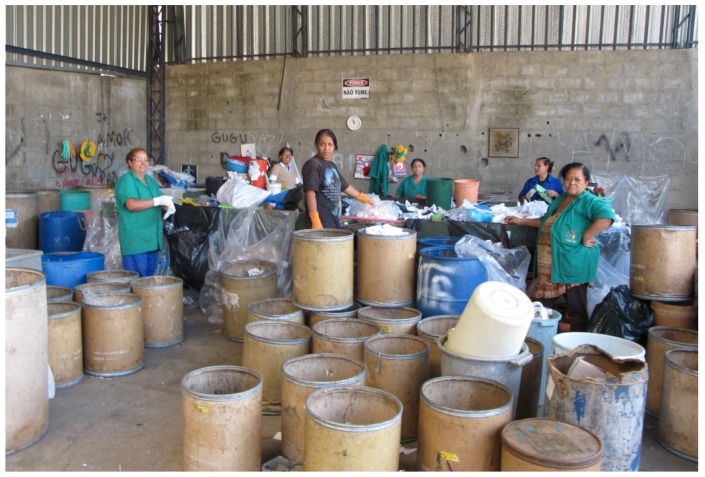
Classification of recyclable material in Diadema.

The recyclers refer to the phase of material entrance to the cooperative as being an occupational health risk due to the dirty, sharp, and contaminated materials they are dealing with. In addition, the recyclers also referred to the heavy weight of the bags and how they easily tip over and fall on them while they place them in piles. The recyclers perceive the sorting of the material as a work intense phase, requiring physical force, attention, and knowledge about the property of the materials (Plate 4).

Often the sorting is performed with the material spread on the floor, forcing the workers to remain squatting or sitting on a small stool or box. During this phase the recyclers have to perform repetitive movements and often lift heavy weights. We observe a risky combination that can lead to the development of skeletal muscle problems such as repetitive strain injuries and deviations of the spine.

Working with piles of cardboard is risky, according to the recyclers, because of the danger of slipping when walking on top of the cardboard spread on the floor. Furthermore, the need to deal with ferrous materials means heavy loads and the risk of cuts and perforations by the material. Plastics, glass and discarded furniture have the danger of biological and chemical contamination (for example: urine in bottles and left over cleaning materials in disposed packages), provoking allergies, infections and respiratory diseases.

Accidents happen when pressing the material, particularly if the press does not come with a protective mechanism preventing the hands from being damaged. Continuously removing the bales from the presses and piling them up leads to muscle strains.

**Plate 5:**
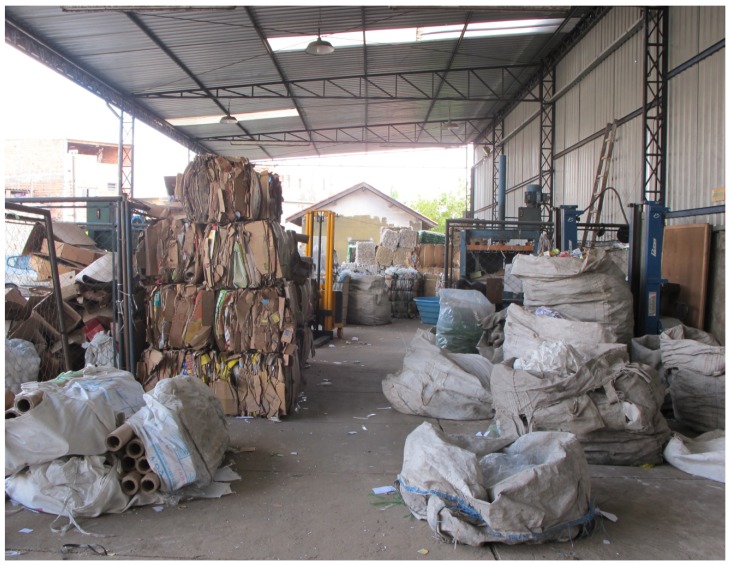
Pressing and bailing of recyclables.

Weighing the bales before selling the material is also not free from occupational hazards, particularly when the balances are old. The activity here involves muscular overload and risk of spinal injury when moving and stacking the bales, which can be an occupational health hazard in the absence of proper transportation within the cooperative. The recyclers repeatedly mentioned how the heavy weight of the bags (with unsorted and sorted materials) and the bales of compressed materials make it difficult to lift, to weight, and to appropriately store them (Plate 5).

Other complaints of the recyclers were unrelated to any particular working phase but were linked to (1) physical space, including inadequate infrastructure, lack of electricity facilities, proper lighting, adequate ventilation, humidity, and water infiltration; (2) biological hazards (presence of mice, cockroaches and pigeons); (3) conflicting human relations, administrative difficulties, and chaotic organization of the work flow and use of the space. In addition to the more apparent occupational health issues such as dermatosis, back problems, and generalized body pain, the recyclers are also subject to poisoning by substances to which they are exposed to during the various phases of the activity, including electrical and electronic components.

We also draw the attention to the use and abuse of alcohol and other drugs by the recyclers, characterized as ‘empty calorie intake’. Fossa and Saad who have researched waste related workers and alcohol consumption affirm: “*(T)he confrontation of the workers’ identity with the lack of value assigned to the activity by the social world can be the generator of this suffering. The feeling of distress may also be linked to the low self-esteem among these workers, some of which just deny their suffering while others compensate it with excessive use of alcoholic beverages*” ([42]: p. 6) (Translation by the authors).

Velloso [43] underlines the level of vulnerability of these workers, due to the lack of structure in their lives, combined with the mental suffering from being exposed to prejudice and aggression in the streets. The recyclers know very well that there are risks related to the precarious situation under which they have to work. They require ways of discharging their frustrations and consequent aggressions, which often includes the consumption of alcohol as a strategy to ease the internal tension.

Santos, seeking to understand the multiplicity of meanings of the work dealing with trash performed in the street exclaims: “*you can say that it (alcoholic beverage) is a metaphor for the process of inclusion by the exclusion of the garbage worker. It replaces the food, energizes the pace, protects against the look of others and masks the shame*” ([44]: p. 169) (translation by the authors).

“*The rum (pinga) is a stimulant to start the hard day’s work and also, to maintain the rhythm imposed by the group. It keeps the workers euphoric and encourages solidarity among them, in addition to addressing the food shortage. ... As ‘bottled medicine’, it cleans your body, which is contaminated by dirt and it eases your work with trash, helping you to not feel like rubbish. The rum is a remedy for everything, mainly to relieve the sorrows and shame of being a catador. The rum cleans the soul and protects the body tainted by exclusion, giving the feeling of inclusion*” ([44]: p. 169) (translation by the authors). Further, Dias [45] observed the living conditions, trajectories, and ways of being a garbage collector and makes reference to the use of illicit drug consumption within this category as being an expression of the roughness of the activity done by these workers.

The research of Nicolau [46] also sees the work as an instrument of social inclusion, particularly when homeless people start working in a recycling cooperative and where social cohesion of the group may help overcome the use of drugs. This author noted that coordinators at the recycling cooperatives developed strategies to prevent the use or to impede the access to drugs, for example, by handing out transportation tickets on a daily basis instead of at the end of the month, thus preventing these tickets from being used as currency for buying drinks.

In more extreme cases, the suffering, humiliation and helplessness of the recyclers is expressed through violence and, finally, mental illness. According to Fossa and Saad: “*…in order to minimize the suffering of these workers and the pleasure optimized, it is necessary not only for the catadores, but by society at large to recognize the importance of the work of selective waste collection and recycling...*” ([42]: p. 8) (translation by the authors). The feelings of disempowerment and vulnerability among recyclers are widespread. Fatalism further reiterates the negative impacts of social exclusion, with the individual accepting inferior treatment and positioning, often restating passive acceptance [40]. These and other authors [47] discuss empathy and the ‘principles of care’ as key strategies to overcome fatalism and to thus reduce occupational risks. It seems that the vicious circle of exclusion has to be interrupted in order to overcome the vulnerability of informal recyclers, particularly those who are struggling with alcohol and drug dependency.

## 4. Waste Management Legislation and Occupational Health: Constraints and Resolutions

The collaborative learning during the workshops and field visits brought in depth knowledge about the critical health concerns in recycling cooperatives. These issues need to be addressed urgently. The government has taken some steps towards improving the conditions of the recyclers and the quality of the work environment at the cooperatives. Particularly since 2002, when the profession of being a *catador* became legalised, several important pieces of legislation have been elaborated on solid waste management, cooperatives, and in particular, with respect to the specific conditions of the *catadores*. The following table (Table 2) summarizes some of the most important public policies and actions of the federal Government in support of resource recovery by *catadores*. Some municipalities have elaborated specific laws in support of the recyclers. In the city of Diadema, for example, the Municipal law 2.336/2004 regulates sustainable waste management and the Decree 5.984/2005 implements the remuneration of the recyclers for the service of collecting and recycling household waste. This is still a pioneering situation for Brazil and abroad, where the service of the recyclers is recognized and valued.

As can be observed in Table 2, the Brazilian government has recently implemented several actions to include cooperatives in the recycling chain and to expand the market of recyclables in Brazil. The fact that the federal law includes solid waste as part of sanitation furthermore extends funding opportunities for this sector. The federal solid waste management policy represents a landmark because it is based on integrated waste management, which means following the principles of prevention and precaution (reduction, reuse and recycling), in addition to applying environmentally appropriate final destination methods. The legislation proposes shared responsibility for the product life cycle and the reduction of negative human and environmental health impacts throughout the life cycle of products.

The Brazilian waste management legislation makes a strong case for defending selective waste collection of reusable and recyclable materials and supporting the *catadores* in that activity. The new law encourages municipalities to hire cooperatives in the selective waste collection and the law promotes studies and research on related topics. Nevertheless, the federal legislation also permits waste incineration when the municipality is unable to implement selective waste collection with organized recyclers. This gap in the legislation consents to a change in the original waste hierarchy, where incineration was allowed only after having exhausted reuse, recycling and landfilling. Recent conflicts have emerged in those municipalities where the local government is prioritizing waste-to-energy, without supporting existing recycling cooperatives or creating new programs and associations to conduct selective waste collection and separation [1,48].

**Table 2:** Key legislation supporting the activity of informal and organized recyclers.

Law/Decree/Action	Main objectives
Federal Law No. 5,764 of December 1971	Establishes the National Policy on Cooperatives
In 2002, the Ministry of Labor and Employment creates the professional category: *catador* ‘collector of recyclable materials’ and includes it in the Brazilian classification of occupations (CBOS), under the Code 5192-05 (MTE. Classificação Brasileira de Ocupações)	Legal and formal recognition of the occupation of collector of recyclable materials, setting parameters for the development of this activity.
Decree No. 5,940, 25 October 2006.	Requires public institutions to separate and donate the recyclable fraction of their solid waste to recycling associations and cooperatives.
Federal Law No. 11,445, of 5 January 2007: National Policy on Basic Sanitation	Authorizes the municipalities to hire recycling associations and cooperatives to collect, process and market recyclable or reusable municipal solid waste.
Federal Law No. 12,017 of August 2009 and published the annex VII of the D.O.U, 13.8.2009, extra Edition.	Changes the law of the budget guidelines, allowing the direct transfer of resources to cooperatives, without intermediation of municipalities or social organizations of public interest.
Federal Law No. 12,305, July 2010 and its regulation through Decree No. 7,404 of December 2010.	Establishes the National Solid Waste Policy and creates the Inter-ministerial Committee of the Brazilian solid waste Policy and the Steering Committee for the implementation of the reverse logistics systems.
Federal Decree No. 7,405, 23 December 2010, published in D.O.U. of 23 December 2010.	Institutes the ‘*Pro-Catador*’ program. It creates the joint inter-ministerial Committee for social and economic inclusion of the collectors of reusable and recyclable material.
Federal Law No. 12,690, of 19 July 2012 published in D.O.U., 20 July 2012.	Rules on the organization and functioning of Workers’ Cooperatives.

The *Pro-Catador* program, for example, takes aim at integrating and coordinating the actions of the Federal Government in supporting and promoting the organization of recyclers, pointing towards better working conditions, more opportunities for social and economic inclusion, expansion of the selective waste collection, reuse, and recycling through the actions of the recyclers.

Even not being specific to the category of *catadores*, the law 12,690, of 19 July 2012, about the organization and functioning of Workers Cooperatives, opens great possibilities for improvements in the workplace of the cooperative. According to Article 8 of this legislation, cooperatives have to follow the same health and safety standards promoted by the existing legislation, and normal acts issued by the competent authorities. The Ministry of Labour and Employment, according to Article 17, has the right to enforce the provisions of this law. From the point of view of occupational health and workers’ safety, until then, there was no legal device in support of this category and very little was discussed or carried out within that framework. The above-mentioned law points to a number of possibilities with respect to the health and safety issues for recyclers, and in our view represents a breakthrough because selective waste collection is no longer an occupation that lacks specific normative actions to promote health and safety at work.

Nevertheless, the narrow and un-contextualized application of this law can become problematic since most cooperatives operate under insufficient sanitary and health conditions. If government officials apply the law without collaborating with cooperatives to reduce their occupational health issues and risks, that could mean closing most of the cooperatives due to insufficient adequacy to the law. Technical support is needed to help cooperatives reorganize their workspace and reduce specific physical, biological, chemical and ergonometric risks as identified by the research [49].

The interactive workshops also allowed the discussion of solutions for the number of serious occupational risks identified by the recyclers. As presented earlier, these risks are related to chemical (e.g., from handling cement bags and containers with toxic products) and biological hazards (e.g., from packaging contaminated by bacteria or fungus, and infections due to cuts from glass, paper, metal or other sharp objects). These problems can be addressed by educating the general population about the quality and cleanliness of their recyclable waste. In addition, it is recommended for the recyclers to wear gloves and to get the tetanus shot. Often, the recyclers mention that with gloves, they lack the tactile perception that is important to identify different types of materials, particularly plastics. For that reason they often don’t like to wear gloves. As a solution, taking off the tips of the thumb and index finger of the glove would provide them with the ability to still identify materials while most of the hand would be protected from contact with sharp objects and contaminated material [50].

Musculoskeletal damage due to performing the separation under inadequate ergonometric and organizational conditions and carrying heavy loads can be reduced by sorting tables in accordance with ergonometric standards and by adopting proper posture when lifting heavier loads if a forklift is not available. Introducing adequate machinery in moving heavy loads is also a measure to increase the effectiveness of the recyclers’ work. An electrical cart for recycling purposes has been designed for door-to-door collection. *Itaipu Binacional*, for example, has developed an electric cart adapted to the necessities of the recyclers, obtainable for the price of approximately 5,000 Brazilian Reals (2,184 US$ as of 11 September 2013). This cart would make a difference in the life of the *catadores* and should be made available.

Mechanical trauma from accidents in the street or at the work place can be addressed with educational measures (e.g., place a maximum of four bales in a single stack) and awareness building, involving the recyclers (promoting organization and cleaning as well as extermination of insects and rats in the cafeteria, locker rooms and restrooms) and the general public (clean material separation at the source). Finally, poor emotional wellbeing due to stress, lack of illumination, and poor air circulation are serious issues. The latter could be solved with changes in the building structure and implementing better lighting. The collaboration of the municipal government is essential to adopt procedures that consider the workers’ health protection in selective waste collection and separation. Finally, Lavoie [51] points out concerns about the growth of recycling-related jobs and, as a consequence, the risks that had not yet been considered. The proposition of health and safety measures to the working environment of these jobs is a new challenge that needs to be included in the political agenda.

## 5. Conclusion: Health Risks and recommendations to Improve Working Conditions for Recyclers

The interactive research process allowed the discovery of a number of serious occupational risks related to chemical, biological and physical hazards, musculoskeletal damage, mechanical trauma and poor emotional wellbeing. In addition, in some cooperatives, frustration and dissatisfaction were the result of a lack of transparency and little participation in the cooperatives’ decision-making. Some participants raised stressful work relations amongst cooperative members as a key health issue and we learned that these were often related to leaders who were emulating hegemonic and oppressive social structures, including unequal gender relations.

It is worth mentioning that there are also “false cooperatives”, which are in fact the reproduction of businesses that mock labor laws to avoid labor incumbencies and that do not respect the international principles of cooperatives as recommended by the International Labor Organization (ILO) in their *Cooperatives Recommendation* Number 193 from 2002 [52] and the International Cooperative Alliance. These “false cooperatives”, for example, do not respect democratic decision making practices while maintaining discriminatory practices; they do not invest in the education of cooperative members, nor do they contribute to the local sustainable development.

Furthermore, the research highlighted the presence of rats, pigeons and insects as serious problems in many of the cooperatives. Local health authorities are aware of these particular problems and regularly spray the cooperative to reduce the infestations. However, the root causes of contaminated recyclables and poor material separation at the source, as well as badly organized work conditions are not yet tackled sufficiently by governmental actions. Continuous environmental education of the public is needed to guarantee a high level of cleanliness of the recyclables.

During several moments of the workshops, acute issues related to social exclusion and homelessness surfaced, escalating with the problems created by alcohol and drug abuse among recyclers, which are usually not professionally treated. The study further revealed emotional health impacts caused by stigmatization and prejudice against the people working with selective waste collection. The recyclers expressed the need to generate more awareness about their work, to become recognized and remunerated as environmental stewards.

The participatory research meant involving the participants early on in the research process. This has allowed for the creation and mobilization of knowledge on health and safety in recycling cooperatives. It has permitted us to collect valuable first-hand information on work related health hazards of recyclers. The multifarious health and safety issues related to the different work activities in the recycling cooperatives were clearly acknowledged and for some of these problems solutions were suggested. Recyclers and researchers alike reiterated that the Government (and particularly the local government) should recognize and address the vulnerable working conditions of recyclers, not through prohibition but by improving the recycling operations and reducing the risks in the cooperatives.

We discussed recent legal advances regarding solid waste and cooperatives and the consequent consolidation of the professional opportunities to improve the work environment of the recycling cooperatives. In the area of solid waste, Brazil has endorsed the national solid waste policy, consolidating the participation of cooperatives as partners in the development of selective waste collection across the country.

Cooperatives represent an alternative economic development model, which is on the rise and is recognized worldwide as a viable path focused on solidarity and social economy. It is not by chance that the United Nations has set the year of 2012 as international year of cooperatives. Cooperatives signify possible interventions, able to bring change in the transition towards a post-capitalist world system. This scenario of informal and organized recycling, particularly in the context of the global south, highlights the importance of this sector and the need for structural and political backing. This also means support for the process of forming new cooperatives, training their members in management, educating on health and safety issues, expanding environmental awareness, investing in communication, as well as strengthening the social and political articulation of the recyclers’ movement together with the movement of homeless people, with reference to the solidarity economy [53].

Ultimately, the collaborative research process has highlighted the complementary nature of academic knowledge to the local knowledge present among the recyclers. Co-generation of knowledge and collective learning provides effective and feasible strategies and resolutions that help tackle acute social and environmental problems, as discussed in this research. It is essential for government health agents to tackle the problematic occupational health situation in recycling cooperatives, by means of constructive interventions reducing health risks and accidents and ultimately improving the quality of life of the recyclers.
